# Tri-Component Hydrogel as Template for Nanocrystalline Hydroxyapatite Deposition Using Alternate Soaking Method for Bone Tissue Engineering Applications

**DOI:** 10.3390/gels9110905

**Published:** 2023-11-16

**Authors:** Irina Mihaela Pelin, Irina Popescu, Manuela Calin, Daniela Rebleanu, Geanina Voicu, Daniela Ionita, Marius-Mihai Zaharia, Marieta Constantin, Gheorghe Fundueanu

**Affiliations:** 1 “Petru Poni” Institute of Macromolecular Chemistry, Grigore Ghica Voda Alley 41A, 700487 Iasi, Romania; impelin@icmpp.ro (I.M.P.); ionita.daniela@icmpp.ro (D.I.); zaharia.marius@icmpp.ro (M.-M.Z.); ghefun@icmpp.ro (G.F.); 2 Institute of Cellular Biology and Pathology “Nicolae Simionescu” of the Romanian Academy, 050568 Bucharest, Romania; manuela.calin@icbp.ro (M.C.); daniela.rebleanu@icbp.ro (D.R.); geanina.voicu@icbp.ro (G.V.)

**Keywords:** chitosan, oxidized pullulan, PVA, calcium phosphates, composite hydrogel

## Abstract

Composite hydrogels containing apatite-like particles can act as scaffolds for osteoblast proliferation, with applications in bone tissue engineering. In this respect, porous biocompatible hydrogels were obtained from chitosan, oxidized pullulan, and PVA in different ratios. The stability of the hydrogels was ensured both by covalent bonds between aldehyde groups of oxidized pullulan and free amino groups of chitosan, and by physical bonds formed during freeze–thaw cycles and lyophilization. The deposition of calcium phosphates was performed by alternate soaking of the porous hydrogels into solutions with calcium and phosphate ions, assuring a basic pH required for hydroxyapatite formation. The mineralized hydrogels were characterized using FTIR spectroscopy, scanning electron microscopy, X-ray diffraction, and thermogravimetric analysis, showing that inorganic particles containing between 80 and 92% hydroxyapatite were deposited in a high amount on the pore walls of the polymeric matrix. The composition of the organic matrix influenced the crystallization of calcium phosphates and the mechanical properties of the composite hydrogels. In vitro biological tests showed that mineralized hydrogels support the proliferation of MG-63 osteoblast-like cells to a greater extent compared to pristine hydrogels.

## 1. Introduction

Bone is a mineralized matrix, a composite containing biopolymers (mostly collagen type I and noncollagenous proteins) and biominerals (biologic apatite—with X-ray diffraction profile as mineral hydroxyapatite) formed by a series of complex reactions orchestrated by different types of bone cells, which interact with each other and with the extracellular matrix [1]. The anatomic and functional continuity of the bone can be interrupted as a consequence of a traumatism or a disease, and depending on the severity of the injury, the restoration of the bone integrity can be performed using various orthopedic implants, bone grafts or synthetic scaffolds [2]. It is very well known that hydroxyapatite is used as coating for inorganic objects, including metallic implants, to enhance their biocompatibility [3,4,5], but to mimic as much as possible the organic–inorganic structure of the bone, synthetic or natural polymers combined with ceramics have been used to provide the best cell integration and bone regeneration [6]. Biomimetic approaches relying on the combination of polymeric hydrogels with apatite-like particles result in composite materials with improved osteomimetic architectures, and osteoconductive and osteoinductive properties, that make them suitable as scaffolds for bone tissue engineering applications [7]. Different products made from collagen sponges and hydroxyapatite are found on the market as bone substitute (Collagraft^®^ (Zimmer and Collagen Corporation), Collapat^®^ II (Ostobalag), Healos (CE Mark & Orquest), Bio-Oss^®^ Collagen (Geistlich), Extra Graft^®^ (Implacil De Bortoli)).

Hydrogels are three-dimensional hydrophilic polymeric networks that can mimic the extracellular matrix and can have biomedical applications in tissue engineering, organs/tissue regeneration, cancer therapy, wound management, drug delivery, or even in vivo diagnostics [8,9,10,11]. The advantage of using hydrogels for bone tissue engineering consists of their biocompatibility and versatile physico-chemical properties: very good elasticity, porosity and water retention ability, which resemble those of the natural collagen matrix from the bone [12,13]. Usually, the polymers able to form hydrogels possess functional groups (carboxylic, sulfonate, phosphate, amine, or hydroxyl groups) that can favor the nucleation and growth of the apatite-like crystals, leading to composite hydrogels [14,15,16,17]. The morphology and the size of the pores (interconnectivity and macroporosity) are important features of hydrogels because they must ensure enough space for osteoblast attachment and proliferation after the nanocrystalline hydroxyapatite (HAp) deposition. Hydrogels with macropores are desirable templates for mineralization as the calcium and phosphate solutions can easily diffuse through the matrix [12]. The functional groups of the hydrogels provide nucleation sites when cationic or anionic solutions are added and apatite nanocrystals are formed via precipitation. Rey et al. explained that the nanocrystals are unstable and upon aging in solutions the proportion of ions in the surface hydrated layer (that cover the nanocrystals) decreases and that of ions in the apatite domain increases [18], which determines the apatite crystals’ growth. Not in the least, the resulting composite hydrogels must favor the transport of growth factors and nutrients necessary for cell migration, survival, differentiation, and proliferation [19].

In bone tissue engineering, hydrogels made from polysaccharides from different sources are more attractive not only because they can better mimic the extracellular matrix, but also because they are considered safer, biodegradable, and do not result in toxic compounds after their biodegradation [20]. Chitosan (CS) is one of the most explored polysaccharides due to its biocompatibility, bioactivity, polycationic nature, intrinsic antibacterial activity, and ability to form porous scaffolds [21]. CS-based scaffolds support the formation of the mineralized bone matrix and the attachment and proliferation of osteoblasts, being proposed for bone tissue engineering [22,23,24,25]. Porous CS scaffolds are usually obtained by freezing and lyophilization, a procedure that also allows their stabilization through physical interactions. However, chemical cross-linking can be used in addition to freezing for a better stability of the matrix [21,24,26]. To avoid the low molecular cross-linker agents that are usually toxic [27], polymers with functional moieties such as aldehyde groups can be used as macromolecular cross-linkers [28]. Different oxidized polysaccharides can be used for this purpose, including oxidized pullulan (OP) [28,29]; its oxidation leads to the desired aldehyde content [30] and it has been proved to interact with CS via the Schiff base reaction [29,31,32]. In addition, pullulan is a microbial-produced biopolymer used in biomedical applications due to its non-immunogenic, non-carcinogenic, and non-mutagenic nature [33]. Natural polymer-based hydrogels have many good biological features, but weak mechanical properties. These limitations can be overcome by combining polysaccharides with synthetic polymers [6,34]. Poly(vinyl alcohol) (PVA) is often used to improve the mechanical properties of biomaterials and it is also biodegradable and biocompatible. Moreover, PVA was the first polymer that was transformed using freeze–thawing (F-T) method into a physically cross-linked hydrogel [34,35,36,37]. CS/PVA hydrogels can be obtained using the F-T procedure at a well-established polymer ratio; however, the increase in the CS ratio determines the deterioration of hydrogel stability. In fact, at a high CS content, the physical interactions between PVA chains are obstructed, the swelling degree of the hydrogel increases followed by partial leakage of CS macromolecules and, therefore, the mechanical properties weaken [38,39]. To overcome these deficiencies, additional cross-linking of CS is required using frequently bi-functional low-molecular chemical cross-linkers [40], dicarboxylic acids [41], or even gamma irradiation [42]. To our knowledge, oxidized polysaccharides have not been used yet as a macromolecular cross-linker in the obtaining of CS/PVA hydrogels. Nevertheless, composite hydrogels obtained using F-T from CS, PVA, and calcium phosphates were proposed as scaffolds for bone-tissue regeneration [43,44,45].

Calcium phosphates (CaP) are used as inorganic components in bone scaffolds because they are found in the natural bone and can bring osteoconduction to the scaffold [46,47]. Hydroxyapatite (Ca_10_(PO_4_)_6_(OH)_2_) is considered the model compound for biological calcium phosphate even if it is not found in the stoichiometric form in the bone composition [48], but rather as calcium-deficient or substituted HAp. These forms can be obtained from amorphous calcium phosphate, dicalcium phosphate dihydrate, octacalcium phosphate, or α and β tricalcium phosphate through a dissolution–reprecipitation mechanism governed by pH, temperature, and the presence of some ions [49,50]. Composite hydrogels containing micro/nanoparticles of CaP (usually HAp) can be obtained by the incorporation of HAp particles into the polymer solution before the hydrogel formation, or by mineralization of the hydrogels after their preparation. In the first approach, pre-formed HAp particles can be dispersed in the polymer solution [43,51] or HAp can be formed by direct precipitation into the hydrogel precursor solution [14,52,53]. In the second approach, the formation of CaP crystals on the surface or inside the hydrogels can be obtained through immersion of the hydrogels in simulated body fluid [12,54], or via alternate soaking of the hydrogels in solutions containing calcium and phosphate salts [55,56,57,58,59]. The last method can be easily used for porous hydrogels and can lead to the incorporation of high amounts of inorganic particles [57].

The main objective of our work was to obtain composite hydrogels with large pores that allow the nucleation and growth of HAp crystals that can further favor the adhesion and proliferation of osteoblasts. In the first step, the hydrogels were obtained from CS, OP, and PVA through a chemical reaction between CS and OP through the formation of Schiff base and by physical cross-linking induced by seven F-T cycles and lyophilization. The ratio between the three polymers was gradually modified to study the influence of each of the components on the hydrogel formation. In the second step, the mineralization of the porous hydrogels was performed using alternant soaking in CaCl_2_/Tris-HCl and Na_2_HPO_4_ aqueous solutions. The deposition of CaP was demonstrated using FTIR spectroscopy, scanning electron microscopy, and thermogravimetric analysis, and the identification of calcium phosphate phases was performed using X-ray diffraction. The mechanical properties of the mineralized hydrogels were compared to those of the pristine hydrogels. The adhesion and proliferation of MG-63 osteoblast cells in the hydrogels were evaluated by visualization of the F-actin fiber distribution after 24 h and 7, 14, and 21 days of cell culture. The cytotoxicity of the hydrogels was also evaluated through quantification of the released adenylate kinase and by using the LIVE/DEAD assay.

## 2. Results and Discussion

### 2.1. Preparation and Preliminary Characterization of the Hydrogels

**Figure 1:**
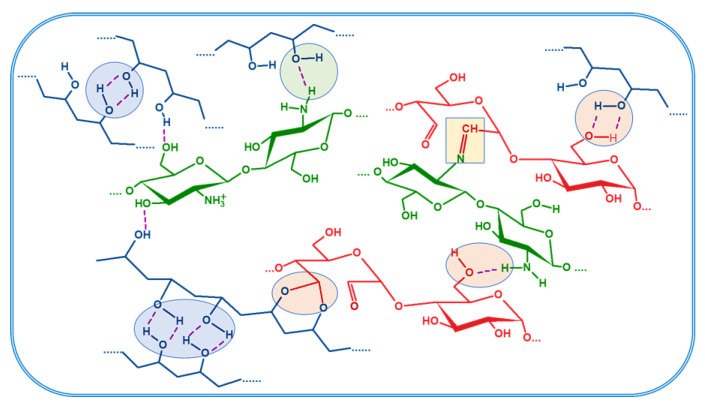
Schematic view of the physical and chemical bonds between the components of the hydrogel (PVA—blue; Chitosan—green; Oxidized pullulan—red).

Tri-component hydrogels containing CS, OP with 30% degree of oxidation, and PVA in different ratios (Table 1) were obtained using the double cross-linking procedure: the Schiff base reaction between the amino groups of CS and aldehyde groups of OP and physical cross-linkings resulted after seven F-T cycles and lyophilization. Hydrogen bonds are formed between PVA chains, PVA and CS, PVA and OP, and CS and OP chains, as presented in Figure 1. Besides these, the hydrogels can also contain acetal or intra-residual hemiacetal structures [31]. Moreover, the freezing process of the PVA solution is also known to induce the formation of micro-crystallites, which act as cross-linking points [37].

To study the influence of each of the three polymers on the properties of the hydrogels, different ratios between them were used. The samples were encoded CS_x_/PVA_y_/OP_z_, where x:y represents the gravimetric ratio between CS and PVA, and x:z represents the molar ratio between amine groups of CS and aldehyde groups of OP, as presented in Table 1. The concentration of CS was maintained 1 wt. % in all the precursor solutions.

**Figure 2:**
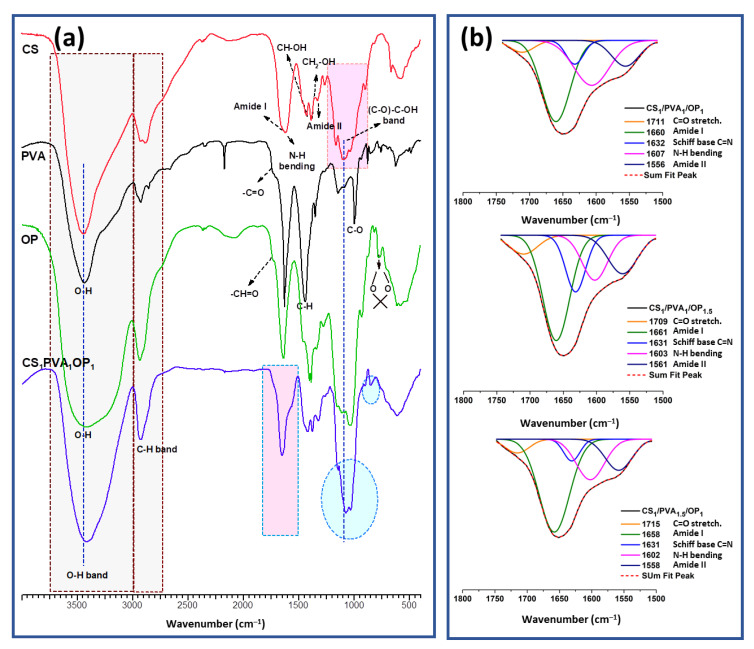
FT-IR spectra of CS, PVA, OP and CS_1_/PVA_1_/OP_1_ hydrogel (**a**) and deconvoluted FT-IR spectra (1800–1500 cm^−1^) of CS/PVA/OP hydrogels (**b**).

The presence of chemical and physical cross-linking bonds in CS/PVA/OP hydrogels was verified using FT-IR spectroscopy. Figure 2 shows the FTIR spectrum of CS_1_/PVA_1_/OP_1_ hydrogel together with the spectra of the polymeric components. In the FT-IR spectrum of CS, the characteristic absorption bands appeared at 1652 cm^−1^ (C=O stretching amide I), 1616 cm^−1^ (N-H stretching amine groups), 1526 cm^−1^ (N-H bending amide II), 1325 cm^−1^ (C-N amide III), 1159 cm^−1^ (C-O-C bridge) and 1092 cm^−1^ (skeletal vibration of C-O) stretching, respectively. The broad band at 3435 cm^−1^ was attributed to -OH and -NH symmetrical vibrations and the peaks at 2874 and 2918 cm^−1^ belonged to -C-H stretching vibrations [52]. The main absorption peaks of pure PVA appeared in the FTIR spectrum at 3412 cm^−1^ (-OH stretching from the inter- and intra-molecular hydrogen bonds) [60], 2862 cm^−1^ (-CH stretching), 1715 cm^−1^ (-C=O stretching non-hydrolyzed vinyl acetate groups), 1419 cm^−1^ (-CH bending) and 1093 cm^−1^ (-C-O- stretching) [61]. The FT-IR spectrum of OP confirmed the successful oxidation of pullulan by the presence of the peak at 1733 cm^−1^, characteristic of the aldehyde group in the dialdehyde pullulan [29,32,33]. Also, the band at 872 cm^−1^ was attributed to the -C-H out-of-plane bending from resultant hemiacetal bonds between aldehyde groups and neighbor hydroxyl groups [62]; the sharper peak at 1035 cm^−1^ owing to C-O-C stretching is a result of the hydration of aldehydes [63]. The FT-IR spectrum of the CS_1_/PVA_1_/OP_1_ hydrogel (Figure 2a) shows all the abovementioned characteristic peaks for pure components. However, there are obviously slight shifts to lower wavenumbers such as at 3435 cm^−1^ (stretching vibrations of hydrogen bonded O-H and N-H in PVA and CS) at 3419 cm^−1^, 1353 cm^−1^ (wagging vibrations of C-H in PVA) at 1325 cm^−1^, 1093 cm^−1^ (stretching C-O in CS and OP) at 1076 cm^−1^, suggesting the existence of H-bonds between hydroxyl groups in PVA and OP and OH and NH_2_ groups in CS chains [64]. In addition, in the 1500–1800 cm^−1^ region, a shoulder appears at 1560 cm^−1^, which can be attributed to the amide II vibrations having shifted to higher frequency due to the formation of H-bonds [65]. Deconvolution of this region (Figure 2b) revealed a peak at 1630 cm^−1^, which is attributed to the imine bond formed due to C=N linkages between CS and OP, confirming the covalent interaction (Schiff-base) in the hydrogel. Additionally, Figure 2b shows that the signals of amide I (1660 cm^−1^) and amide II (1560 cm^−1^) bands increased with the increase in the CS amount (CS_1_/PVA_1_/OP_1.5_ and CS_1_/PVA_1.5_/OP_1_) and the signals of the NH_2_-free groups (about 1600 cm^−1^) decrease with the increase in the cross-linking degree in CS_1_/PVA_1_/OP_1.5_.

The main features of the purified hydrogels obtained with different ratios between the components are presented in Table 1. The gel fraction (GF) gives information about the amount of cross-linked hydrogels that remained after the washing and purification step. GF ranged between 61 and 67%, values expected for PVA/CS hydrogels obtained using the F-T method [40,41]. The highest GF was obtained for the sample with the highest amount of PVA (CS_1_/PVA_1.5_/OP_1_), showing that the physical cross-linking led to a better maintenance of PVA chains in the hydrogel structure after the washing step. The higher cross-linking degree of the CS (through imine bonds with OP) and the higher content of CS in the sample CS_1_/PVA_1_/OP_1.5_ also suggest that chemical cross-linking plays an important role in the formation of the hydrogels. Comparing the composition of the purified hydrogels with the composition of the precursor mixture, it can be concluded that the excess of OP was removed from the hydrogels. Most probably, this is due to the relatively low molecular mass of OP [32]. A lower ratio of PVA was found in the composition of the purified CS_1_/PVA_1_/OP_1.5_ sample, showing that the high concentration of OP impedes the physical cross-linking of PVA.

**Figure 3:**
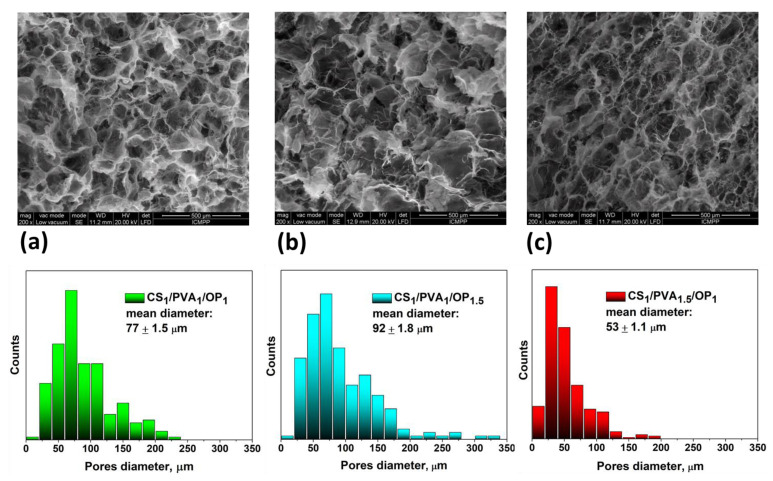
Morphology and size distribution of the pore diameter of the CS_1_/PVA_1_/OP_1_ (**a**), CS_1_/PVA_1_/OP_1.5_ (**b**) and CS_1_/PVA_1.5_/OP_1_ (**c**) hydrogels.

It is known that the F-T method leads to the obtaining of macroporous hydrogels with interconnected pores. The size of the pores is an important feature of the matrices designed to be used in bone tissue engineering [6,7], especially if the introduction of CaP is intended to be performed using alternant soaking. In this respect, the morphology of the hydrogels was investigated using SEM. Figure 3 shows the SEM micrographs of the fractured central zone of the hydrogels after purification and lyophilization, together with the pore size distribution depicted underneath. The image of the CS_1_/PVA_1_/OP_1_ hydrogel (Figure 3a) reveals distinctive pores, typically resulting from the F-T method, with a mean diameter of 77 µm and pore walls that seem thicker compared to the other two hydrogels. When the amount of OP was increased (CS_1_/PVA_1_/OP_1.5_ sample), a porous microstructure was observed, with the largest size distribution of the pores and a mean diameter of 92 µm (Figure 3b). This confirms our supposition that OP in high amounts impedes the physical cross-linking of PVA. The walls of the pores are not as well defined as in the CS_1_/PVA_1_/OP_1_ hydrogel. In the case of the CS_1_/PVA_1.5_/OP_1_ hydrogel, the pore distribution is more uniform and the medium pore size decreased to around 53 µm due to the physical cross-linking of PVA found in high amounts in this sample. The thickness of the walls seemed to be lowest in this case compared with the other two samples. It is expected that these differences in the morphology of the hydrogels will influence the deposition of CaP.

### 2.2. Synthesis and Characterization of Composite Hydrogels Containing CaP

**Figure 4:**
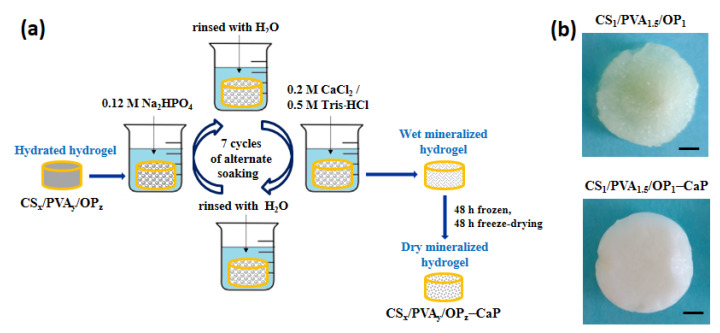
(**a**) Preparation steps of mineralized hydrogels; (**b**) macroscopic images of the CS_1_/PVA_1.5_/OP_1_ and CS_1_/PVA_1.5_/OP_1_-CaP hydrogels (scale bar: 2 mm).

The mineralization of the hydrogels consisted of alternating immersions of the hydrogels in Na_2_HPO_4_ and CaCl_2_/Tris-HCl solutions (Figure 4a), with a pH of around 7.4 to ensure CaP formation and deposition. Some papers have reported that the metastable polymorphs (amorphous calcium phosphate, dicalcium phosphate dihydrate—DCPD, and octacalcium phosphate) transform into stable apatite under physiological conditions [66,67,68]. Other papers focused on CaP pre-nucleation clusters, which are calcium triphosphate ion-association complexes that further lead to the nucleation of amorphous calcium phosphate, and noted that by continued calcium uptake this is converted into octacalcium phosphate and then into highly substituted apatite [69,70,71,72]. Rey et al. [18,48] highlighted the existence of a structured hydrated layer on the surface of the HAp nanocrystals, which progressively transformed into the more stable apatitic lattice upon aging in aqueous media, and demonstrated that loosely bound ions of the hydrated layer can be easily and reversibly substituted by other ions in fast aqueous ion exchange reactions. Also, it was observed that during maturation, the ions gradually fill crystallographic positions corresponding to the apatitic lattice, leading to the increase in the Ca/P ratio and indicating an evolution toward stoichiometric HAp [73,74].

In our experiments, we started from the supposition that functional groups of the polymers would be favorable for in situ formation of bone-like apatite by successive immersions of the hydrogels into ionic phosphate and calcium solutions. Between the immersions, the hydrogels were washed with water to remove the excess of Na_2_HPO_4_ and CaCl_2_. The maturation of the hydrogels during the nights at room temperature at slightly alkaline pH, in phosphate or calcium solution, ensured the transformation of metastable polymorphs of calcium phosphate initially formed into nanocrystalline HAp [68]. Since the exact structure of the inorganic particles was not known after mineralization, the composite samples were noted as CS/PVA/OP-CaP. Figure 4b shows the macroscopic images of the hydrated hydrogel before and after mineralization, when the CaP deposition was clearly observed: the color of the CS_1_/PVA_1.5_/OP_1_ hydrogel changed from pale yellow to white.

**Figure 5:**
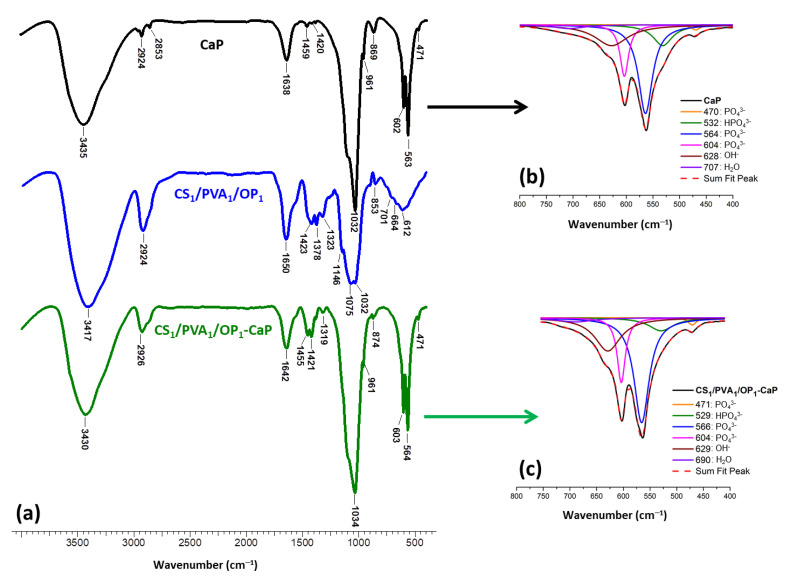
FT-IR spectra of CaP, CS_1_/PVA_1_/OP_1_, and CS_1_/PVA_1_/OP_1_-CaP hydrogel (**a**) and deconvoluted FT-IR spectra (400–750 cm^−1^) of CaP (**b**) and CS_1_/PVA_1_/OP_1_-CaP hydrogel (**c**).

The deposition of CaP onto/into hydrogel was confirmed using FT-IR spectroscopy. Figure 5 presents the spectrum of mineralized hydrogel CS_1_/PVA_1_/OP_1_-CaP together with the spectrum of the pristine hydrogel and that of CaP formed in the absence of the organic template. The IR absorption bands from the spectrum of CaP are close to the characteristic nanocrystalline HAp [73,74]: the shoulder around 1091 cm^−1^ and the stronger peak at 1034 cm^−1^ correspond to the ν_3_(PO_4_^3−^) asymmetric mode, the peak at 961 cm^–1^ were assigned to ν_1_(PO_4_^3−^), and the peaks at 603 and 564 cm^–1^ were attributed to ν_4_(PO_4_^3−^). From the deconvolution of the large band from 750–400 cm^−1^ (Figure 5b), other peaks can be observed: at 628 cm^−1^ corresponding to apatitic OH^−^, at 532 cm^−1^ attributed to “non-apatitic” HPO_4_^3−^, and at 470 cm^−1^ attributed to ν_2_(PO_4_^3−^) [73,74]. The peak at 1638 cm^−1^ was assigned to H–O–H bending from physically and chemically adsorbed water, characteristic of HAp obtained through wet precipitation [75]. The small peaks at 1459 and 1420 cm^−1^ correspond to traces of ν_3a_ (CO_3_^2−^) B-type carbonated apatite [76]. Compared to the pristine hydrogel, in the spectrum of mineralized hydrogel the occurrence of CaP can be observed by the presence of the peaks characteristic to phosphate groups located in regular apatite crystallographic sites: 961, 604, and 564 cm^−1^. The significant increase in the peak from 1034 cm^−1^ is also due to the phosphate from HAp. The signal of the “non-apatitic” phosphate ions located on the surface of the nanocrystals was shifted from 532 cm^−1^ in CaP to 529 cm^−1^ in the mineralized hydrogel due to the interference of the polymers with the hydrated layer of the nanocrystals.

**Figure 6:**
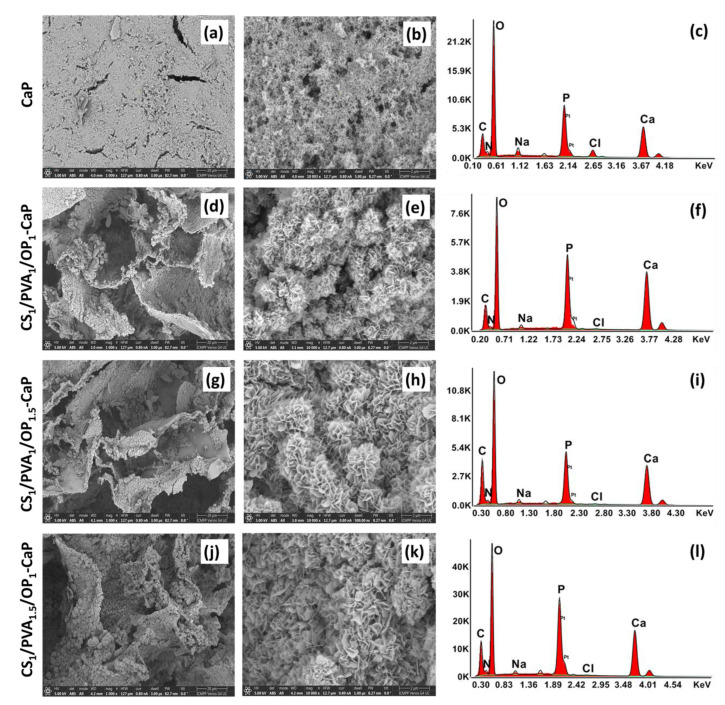
SEM images at two magnifications (1000× and 10,000×) and EDX analysis of the CaP obtained in the absence of hydrogel (**a**–**c**) and CaP deposition onto pore walls of the hydrogels: CS_1_/PVA_1_/OP_1_-CaP (**d**–**f**), CS_1_/PVA_1_/OP_1.5_-CaP (**g**–**i**), and CS_1_/PVA_1.5_/OP_1_-CaP (**j**–**l**).

The SEM images of the cross-section of the mineralized hydrogels (Figure 6) also proved the deposition of CaP on the walls of the polymeric network of all hydrogels. The crystallites appear to have a lamellar morphology, suggesting the transformation of metastable polymorphs of CaP with polygonal morphology into apatite structures [49]. These lamellar nano-flakes formed florette-like particles, as in the case of other hydrogels mineralized by alternant soaking [56,57,58]. These “rosette” structures can be explained by the nucleation of apatite from different locations on the surface of the pore walls and the formation of crystallites outwards in a radial pattern [57]. From the SEM images, it can be observed that in the case of CS_1_/PVA_1_/OP_1_-CaP hydrogel, the size of florette-like particles seems the smallest, while the hydrogel with the highest ratio of PVA (CS_1_/PVA_1.5_/OP_1_-CaP) has the highest dimension of the particles. This suggests a higher number of nucleation sites in the case of the CS_1_/PVA_1_/OP_1_ hydrogel, which also presented the smaller size of the lamellar apatite flakes. It is known that the chelation of amino groups in CS with calcium ions may facilitate the nucleation of apatite [58].

SEM-EDX analysis was used to determine the elemental composition of the deposited CaP in mineralized hydrogels, and the Ca/P ratios are presented in Table 2. For pure HAP, the Ca/P ratio is 1.67, but for other calcium phosphate polymorphs, this ratio is usually lower than 1.67. In the case of CS_1_/PVA_1.5_/OP_1_-CaP, the Ca/P ratio was close to 1.67, but for the other two hydrogels, this ratio was higher. The last two hydrogels presented a higher ratio of CS compared with CS_1_/PVA_1.5_/OP_1_, and CS is known to have a high chelating ability for metal ions, and also for Ca^2+^ [77]. That is probably why calcium ions were found in excess in the mineralized hydrogels.

**Figure 7:**
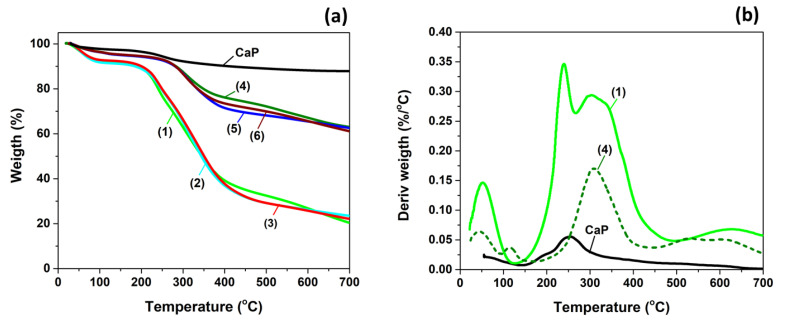
(**a**) TG curves for CaP and hydrogels before and after mineralization and (**b**) DTG curves for CaP, un-mineralized, and mineralized CS_1_/PVA_1_/OP_1_ hydrogel. (1—CS_1_/PVA_1_/OP_1_; 2—CS_1_/PVA_1_/OP_1.5_; 3—CS_1_/PVA_1.5_/OP_1_; 4—CS_1_/PVA_1_/OP_1_-CaP; 5—CS_1_/PVA_1_/OP_1.5_-CaP; 6—CS_1_/PVA_1.5_/OP_1_-CaP).

The ratio between organic and inorganic components in mineralized hydrogels was determined using thermogravimetric analysis (TGA). Figure 7a shows the thermograms of CaP formed in the absence of the organic matrix, and of the hydrogels before and after mineralization. The inorganic CaP loses the physically adsorbed water between 60 and 200 °C and the lattice water molecules between 200 and 300 °C. Above 300 °C, the conversion of calcium hydrogen phosphate to calcium pyrophosphate leads to water release [78,79,80]. However, a residue of 88% remained from the inorganic CaP at 700 °C.

The CS/PVA/OP hydrogels have around 9% humidity and no visible differences can be observed in the degradation curves of the hydrogels with different compositions. The PVA/CS hydrogels obtained using F-T are known to have a principal decomposition stage between 265 and 430 °C [81]. In our case, an additional degradation stage can be observed between 210 and 250 °C (Figure 7b) due to the presence of OP in the hydrogels. In the thermogram of OP a thermal degradation stage between 220 and 250 °C was observed before the principal degradation stage (250–400 °C); it is known that for oxidized polysaccharides with aldehyde groups, the main decomposition stage has a double peak and the onset of the thermal degradation is lower compared to the parent polymers [82,83,84].

The mineralized hydrogels are thermally more stable compared to the pristine hydrogels, the onset of the main decomposition stage being around 265 °C. They also present a much smaller weight loss due to the high amount of deposited inorganic components. From the residue amount at 700 °C, the mass fraction of deposited CaP was calculated assuming a negligible interaction between organic and inorganic phases during thermal decomposition. The results presented in Table 2 show that all the mineralized hydrogels contain around 63% CaP, meaning that a high amount was deposited using the alternant soaking method, but the ratio between the polymers does not influence this amount.

**Figure 8:**
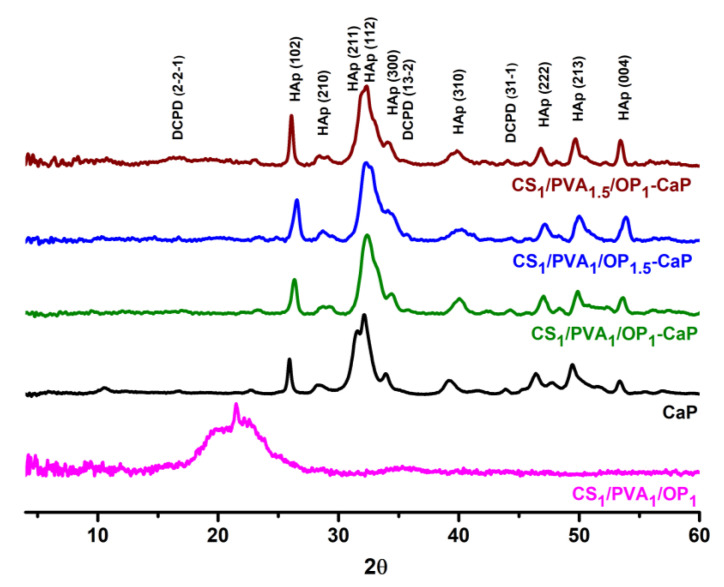
XRD patterns of the CS_1_/PVA_1_/OP_1_ hydrogel, CaP and mineralized hydrogels.

XRD spectra were recorded to investigate whether the crystallographic structure of CaP from the mineralized hydrogels was HAp or other non-apatitic phases. In Figure 8, the XRD patterns of the pristine hydrogels, CaP obtained in the absence of organic matrix, and composite hydrogels are presented. The CS_1_/PVA_1_/OP_1_ hydrogel presents a broad diffraction peak at 2θ around 20°, a peak found in PVA hydrogels obtained using F-T [85], or in PVA/CS hydrogels [40,41] and characteristic for both CS and PVA. The broadening of this peak is due to the amorphous structure of CS [86].

In the literature, it has been reported that the X-ray diffraction pattern of the CaP and mineralized hydrogel corresponds to that of HAp with low crystallinity [16,50,73,74]. Moreover, the XRD patterns of the mineralized hydrogels are very close to the pattern of human bone [87]. The characteristic peaks of PVA and CS from the hydrogel were not observed in the XRD spectra of the mineralized hydrogels due to the very large quantity of deposited CaP. Apart from nanocrystalline HAp, the diffraction peaks corresponding to the non-apatitic phase (DCPD) were also found in the patterns of CaP and the mineralized hydrogel. It was proved that DCPD is formed in the wet precipitation of CaP, which is converted into HAp during maturation in an alkaline medium [67,68]. On the other hand, this non-apatitic form does not fully convert into HAp under normal pressure and temperature conditions [59,68], so DCPD was also found in the mineralized hydrogels (Figure 8).

Table 3 presents the quantitative analysis of the samples and the main crystallographic features: Miller indices, crystallite size, crystallinity, and lattice volume. Whole Powder Pattern Fitting (WPPF) parameters Rwp (<10) and S (around 1), as well as χ^2^ values, support the statement made above. Characteristic (hkl) Miller indices (102), (210) at around 26°, (211) at 33°, and (300), (310), (222), (213), and (004) in the XRD spectra of mineralized hydrogels, were comparable with existing cif data (S around 1) from the Crystallography Open Database (COD-9002214). The results reveal that the HAp crystals formed in the presence of the tri-component hydrogel belonged to the hexagonal space group P63/m, with cell constants close to the values reported in the literature: a = b = 9.40 Å and c = 6.813 Å; α = β = 90°; and γ = 120° [16,74].

As the data from Table 3 also show, the yield of HAp formed in the presence of hydrogels was higher comparatively than that from CaP formed in the absence of the polymeric matrix. The hydroxyl polar groups favor the nucleation and the growth of nanocrystalline HAp whether they belong to polysaccharides or PVA chains [53]; the amino groups of CS also facilitate apatite nucleation [58,88,89]. The decreasing percentage of DCPD from 21% in CS_1_/PVA_1_/OP_1_-CaP to 11% and 8.5% in the other two composite samples is difficult to explain because it cannot be correlated only with functional groups of the polymers. The different pore interconnectivity and the thickness of the pores’ walls in the hydrogels can influence the contact between the calcium/phosphate solutions and the organic matrix, which will further influence the growth of the inorganic crystals. This supposition is more evident in the case of CS_1_/PVA_1.5_/OP_1_ hydrogel, which had the lowest porosity and thinner walls, meaning an increased contact with the precursor solution and with amorphous CaP and leading to a higher transformation of DCPD into HAp. Anyway, the presence of DCPD has been proven to participate in new bone formation when implanted alone or in other composite materials into bone defects in animal models [90,91,92].

To investigate the properties required for bone-like scaffolds, the swelling capacity, the mechanical properties and the cytocompatibility of the mineralized scaffolds were measured.

**Figure 9:**
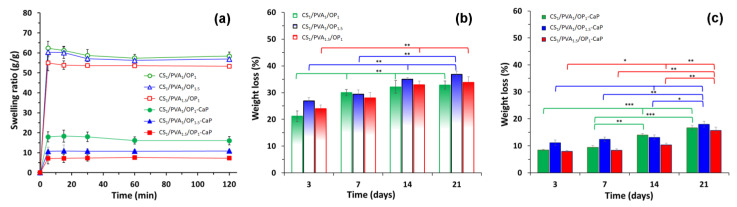
Swelling ratio (**a**) and mass loss of the hydrogels without (**b**) and with CaP (**c**) in simulated physiological conditions (PB pH = 7.4, 37 °C). Results are expressed as means ± standard deviation (S.D.) of three (n = 3) experiments: * *p* < 0.05, ** *p* < 0.01, *** *p* < 0.001..

The swelling behavior of the freeze-dried hydrogels without and with CaP was evaluated by soaking in phosphate buffered with pH = 7.4 at 37 °C. The swelling kinetics presented in Figure 9a are characteristic of hydrogels obtained using the F-T method with high pore size and pore interconnectivity [40,44,93]: a fast swelling was observed in the first five minutes, and equilibrium was reached in the first hour. The samples with a high content of CS, CS_1_/PVA_1_/OP_1_, and CS_1_/PVA_1_/OP_1.5_ had a higher swelling ratio due to the hydrophilicity of the polysaccharides. The hydrogels with the higher ratio of PVA and the lowest porosity had a decreased swelling ratio.

It is known from the literature that the addition of CaP into hydrogels decreases their swelling capacity [94], but in the case of CS/PVA/OP hydrogels, the swelling ratio decreased drastically after mineralization (Figure 9a). The deposited CaP in a high amount (63% from the composite weight) covers the walls of the pores, decreasing the volume of the pores and contributing at the same time to the increase in weight of the dry hydrogel without an impact on water retention, leading to the decrease in the swelling ratio. The mineralized hydrogels were arranged in the same order regarding their swelling, as the pristine hydrogels.

The hydrolytic degradation in PBS at 37 °C of the hydrogels was studied in vitro using gravimetric analysis. Figure 9b and c showed an increase in the weight loss of the samples with time, with the degradation rate of the mineralized CS/PVA/OP hydrogels being significantly lower than the CS/PVA/OP ones (*p* < 0.001). However, after 21 days, the CS/PVA/OP samples lost only 37% of their initial weight due to the presence of covalent cross-linking of the CS and OP chains (see Table 2). In the first 3 days, the 20 ÷ 28% mass loss of un-mineralized hydrogels compared with only 8 ÷ 11% for CS/PVA/OP-CaP could be attributed to the hydrolysis and rapid diffusion of a small fraction of less-crosslinked polymeric components. The hydrogel CS_1_/PVA_1_/OP_1.5_ showed a slightly higher weight loss owing to the presence of a higher amount of polysaccharides and a more porous structure. Increasing the PVA content in CS_1_/PVA_1.5_/OP_1_ resulted in more stable hydrogels. Overall, since the hydrolysis of Schiff’s base is very low under physiological conditions, CS/PVA/OP hydrogels showed good stability. The stability of HAp particles at pH 7.4 [95] gave a more reliable consistency of the mineralized CS/PVA/OP-CaP hydrogels (Figure 9c). In addition, the deposition of HAp on the pore walls can also protect the polymeric matrix from hydrolysis.

**Figure 10:**
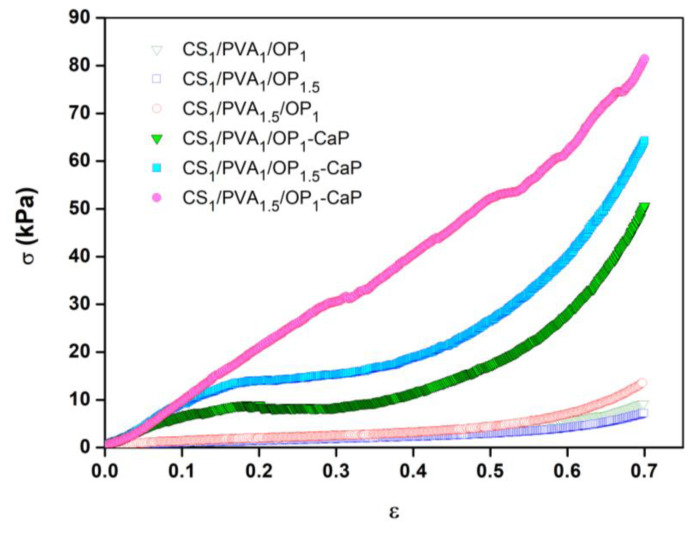
The stress–strain compression curves of the un-mineralized and mineralized hydrogels in the swelling state.

The uniaxial compressive tests were used to evidence the influence of mineralization on the mechanical properties of hydrogels. The compressive stress (σ)–strain (ε) curves of the swollen hydrogels are presented in Figure 10. The CS/PVA/OP hydrogels without CaP presented a typical behavior for porous hydrogels obtained using F-T from PVA [96] or PVA/polysaccharide [40,93,97] solutions with a low polymer concentration. For these porous scaffolds, a large deformation was obtained when low stress was applied (linear region), but beyond 50–60% deformation, after the compression of the pores, the compressed material became dense, and higher stress was required to obtain a deformation (densification region). The elastic modulus and the compressive nominal stress were low for the un-mineralized hydrogels, as presented in Table 2. The variation of the ratio between the three polymers CS, PVA, and OP had a relatively low influence on the hydrogels’ mechanical properties. The sample CS_1_/PVA_1.5_/OP_1_ seemed to have higher mechanical properties due to the denser structure compared with the other two hydrogels (Figure 3), while the sample CS_1_/PVA_1_/OP_1.5_ had the lowest mechanical properties due to the low ratio of PVA in the purified hydrogel (see Table 1).

The compression curves for the mineralized hydrogels are very different compared to the pristine hydrogels (Figure 10) due to the reinforcement effect of the CaP particles. The hydrogels became stiffer, as shown by the increase in the compression modulus (Table 2), and had a much higher toughness. It is known that the introduction of CaP through the mineralization of porous hydrogels/aerogels leads to an increase in the compression stress [94,98,99], but in the case of CS/PVA/OP-CaP hydrogels, this increase was between 400 and 900%, probably due to the high amount of CaP (around 63%) deposited onto the soft polymeric network. The shape of the compression curves suggests that the mineralized hydrogels presented irreversible deformation due to the failure of the inorganic skeleton deposited onto the pore walls, made from HAp lamellar nano-flakes organized in florette-like particles. For CS_1_/PVA_1_/OP_1_-CaP and CS_1_/PVA_1_/OP_1.5_-CaP hydrogels, the failure was observed at around 20% deformation, but no visible fractures were observed until 70% compression strain. The CS_1_/PVA_1.5_/OP_1_-CaP hydrogel has higher mechanical properties due to the higher PVA content, and probably due to the higher size of the CaP particles. In this hydrogel, the Hap/DCPD ratio is the highest, and HAp is known to have better mechanical properties compared to DCPD [100].

### 2.3. Cell Proliferation and Cytotoxic Evaluation

The cytocompatibility of the un-mineralized and mineralized hydrogels was investigated using MG-63 osteoblast-like cells. Since materials containing CS, PVA, OP, and HAp were proved to support the adhesion and proliferation of the bone cells [45,51,53,66,89], it was expected that both the un-mineralized and mineralized hydrogels obtained in our work would be biocompatible and able to be used as matrix in bone tissue engineering. In this respect, the biological markers were evaluated for each seeded hydrogel after 24 h and 7, 14, and 21 days of culture cell.

**Figure 11:**
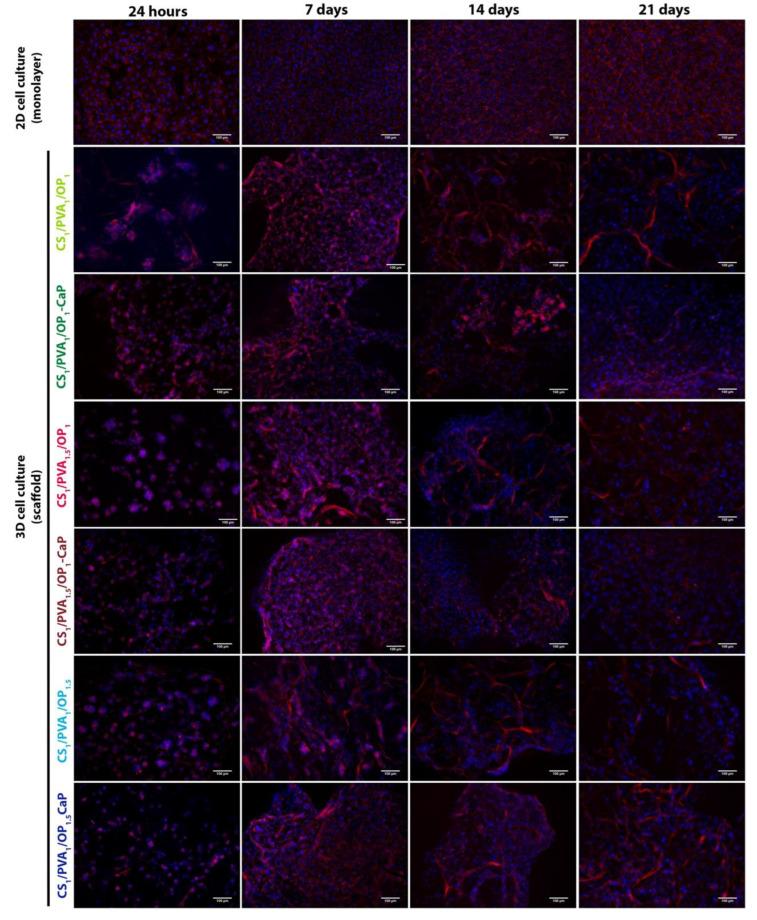
Fluorescence images showing the MG-63 cell morphology by staining the actin cytoskeleton with Phalloidin-TRITC (red) and nuclei with DAPI (blue). Scale bar: 100 µm.

The distribution of F-actin fibers was observed at different time intervals in both types of hydrogel and 2D cultured MG-63 cells (Figure 11). At 24 h after seeding, osteoblasts started to infiltrate the hydrogel and were arranged as clusters, with a much higher number of cells in the mineralized hydrogels. F-actin filaments were visible in cells from clusters arranged along the fibers of hydrogels. Seven days after seeding, we observed that cells proliferated more in the mineralized hydrogels compared with hydrogels without CaP. After 14 days in culture, osteoblasts began to lose the organization of F-actin in the fibers. The seeding of osteoblasts in CS_1_/PVA_1_/OP_1_ and the mineralized hydrogels ensure the organization of F-actin in fibers for up to 21 days.

While mineralized hydrogels support cell proliferation, cells cultured in pristine hydrogels merge from the hydrogel and localize to the culture plate, where they proliferate and form F-actin fibers.

**Figure 12:**
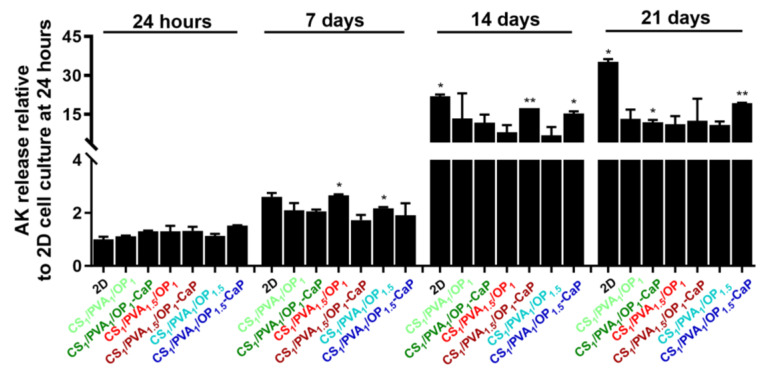
AK released from MG-63 osteoblasts cultured in hydrogels without and with CaP. Data are presented as mean ± S.D. of two experiments performed in duplicates (n = 4) and analyzed using unpaired two-tailed Student’s *t*-test: * *p* < 0.05, ** *p* < 0.01 versus the value obtained on the 2D culture model at 24 h, considered 1.

At 24 h after seeding, adenylate kinase (AK) released from MG-63 osteoblasts cultured in hydrogels showed a statistically insignificant 1.3-fold increase in the CS_1_/PVA_1.5_/OP_1_, CS_1_/PVA_1_/OP_1_-CaP and CS_1_/PVA_1.5_/OP_1_-CaP hydrogels, and a 1.5-fold increase in the CS_1_/PVA_1_/OP_1.5_-CaP hydrogel compared to cells cultured in 2D, where the AK level was considered 1 (Figure 12). After 7 days of culture, the concentration of AK released by osteoblasts cultured in hydrogels was lower than that determined in 2D cell culture, demonstrating the cytocompatibility of the hydrogel for MG-63 osteoblasts. After 14 days of seeding, without passing, the number of viable osteoblasts decreased in both the 2D and 3D culture models, the AK release being much higher compared to that released at shorter time intervals. However, the concentration of AK released from cells seeded in hydrogels was lower compared to cells cultured on 2D media, especially in the case of CS_1_/PVA_1.5_/OP_1_ and CS_1_/PVA_1_/OP_1.5_. While osteoblasts cultured in 2D for 21 days lose viability, with high released AK values, the mortality rate of osteoblasts cultured in 3D models is lower, with AK values remaining approximately constant between different hydrogel types, with no statistically significant changes.

**Figure 13:**
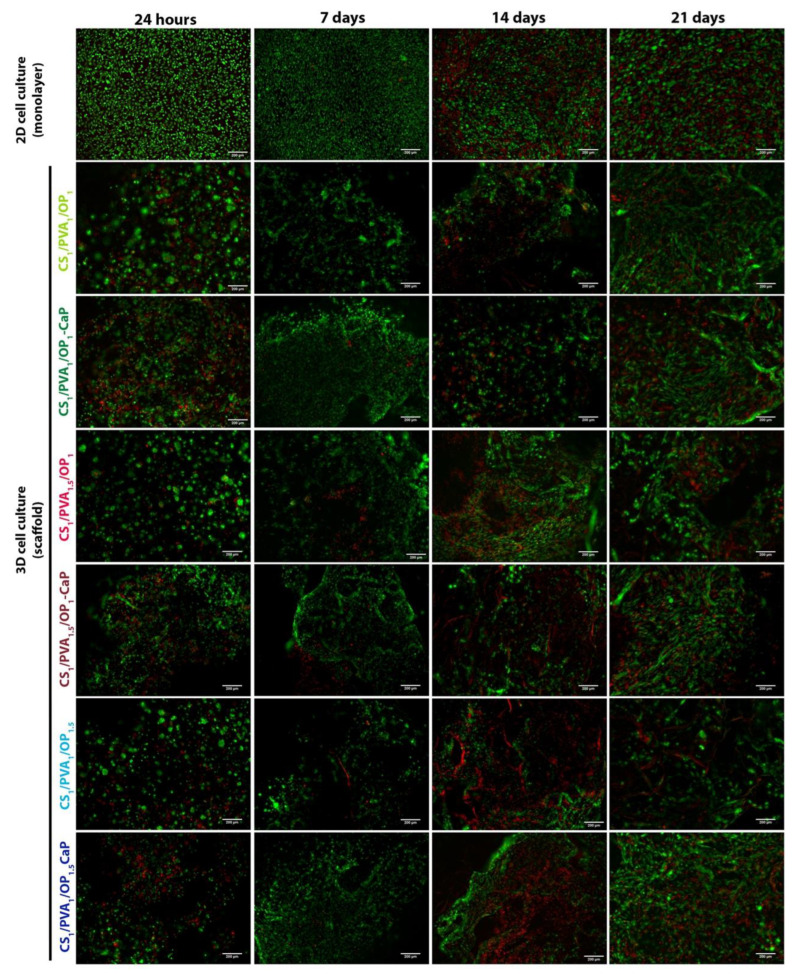
Live (green)/dead (red) assay of MG-63 cells grown in 2D monolayer or in 3D hydrogels without and with CaP. Scale bar: 200 µm.

These results were confirmed by the LIVE/DEAD assay, which stains cells with Calcein/Propidium Iodide to differentiate viable (green) from dead (red) cells (Figure 13). It can be seen that although there was a higher mortality of osteoblasts cultured in hydrogels compared to the 2D model at 24 h, after 7 days, the number of viable cells was increased in the 3D culture models, supporting cell adhesion and proliferation.

**Figure 14:**
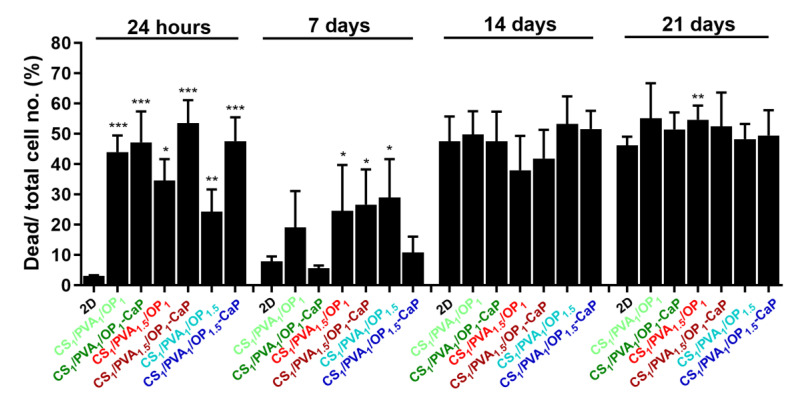
The percentage of dead (red fluorescence)/total cell number (red + green fluorescence) for MG-63 cells grown in 2D culture and hydrogels without and with CaP. These were obtained from live/dead cell assay using ImageJ software version 1.8.0 (U. S. National Institutes of Health, Bethesda, MD, USA) to quantify red and green fluorescence. Data are presented as mean ± S.D. of three fields per probe (n = 6), analyzed using unpaired two-tailed Student’s *t*-test: * *p* < 0.05, ** *p* < 0.01, *** *p* < 0.001 versus the value obtained on the 2D culture model for each experimental time point.

After 24 h of cell seeding, the cells’ viability was lower than expected in the hydrogels containing CaP (Figure 14), probably because of the abundance of the florette-like HAp particles inside the pores of the hydrogels, which can impede the adhesion of the cells in the first day of culture. Shi et al. studied the size effect of nano-HAp particles on MG-63 cells and concluded that the nanoparticles with a size around 20 nm promoted cell growth and inhibited cell death compared to particles larger than 80 nm [101]. However, after 7 days of culture in both un-mineralized and mineralized hydrogels, the cell viability increased remarkably, with the highest values of cell viability observed in the case of the CS_1_/PVA_1_/OP_1_-CaP and CS_1_/PVA_1_/OP_1.5_-CaP hydrogels, which contained, besides CaP nanoparticles, a lesser amount of PVA.

Although after 14 days in culture, the number of viable cells decreased in both culture models, it remained constant up to 21 days in the studied hydrogels compared to the 2D model, where there was a higher number of dead cells. The much lower number of viable (green) cells in 3D models at longer times can be explained by the migration of cells from hydrogels into the culture plate. In 2D cell culture, the high number of dead (red) cells is due to the maintenance in culture for long periods without trypsinization, which resulted in increased osteoblast mortality (Figure 13 and Figure 14).

## 3. Conclusions

Porous tri-component hydrogels were prepared using CS, PVA, and OP in different ratios. The hydrogels were double-cross-linked by covalent imine bonds resulting between aldehyde groups of OP and free amino groups of CS and by physical bonds formed during F-T cycles and lyophilization. The SEM investigation showed that the hydrogel morphology was influenced not only by the presence of the PVA in different amounts but also by the percentage of OP used for the cross-linking of CS.

The porous hydrogels were mineralized through alternant soaking into ionic calcium and phosphate solutions to favor the nanocrystalline HAp formation and deposition on the walls of the pores. TGA investigation showed that all mineralized hydrogels contain around 63% CaP (HAp, DCPD), meaning that using the alternant soaking method, a large amount of inorganic particles were deposited, independent of the ratio between the polymers and the size of the pores. XRD analysis revealed that the CS_1_/PVA_1.5_/OP_1_-CaP hydrogel contained the highest HAp:DCPD ratio, explaining its increased mechanical properties. No significant differences between hydrogels were noticed after MG-63 osteoblast cell culture and cytotoxic evaluation, with the observation that the mineralized hydrogels better support cell proliferation.

The prepared CS/PVA/OP-CaP composite hydrogels showed potential as scaffolds for bone tissue engineering. However, further studies regarding the optimization of the mineralization protocol to demonstrate the osteoconductive potential of the CS/PVA/OP-CaP hydrogels need to be performed.

## 4. Materials and Methods

### 4.1. Materials

Low-molecular-weight chitosan (CS) was purchased from Sigma Aldrich (Steinheim, Germany) and was used as received. The exchange capacity found from conductometric titration using 0.1 M NaOH was 5.25 meq./g corresponding to a 81.26% deacetylation degree. The molecular weight of the CS was 330 kDa, calculated from viscometric measurements [102]. Pharmaceutical pullulan with Mw = 200,000 g/mol was purchased from Hayashibara Lab. Ltd. (Okoyama, Japan). Mowiol^®^ PVA 10–98 with a Mw = 61,000 g/mol and a degree of hydrolysis of 98.0–98.8 mol% was purchased from Sigma Aldrich (Steinheim, Germany). Calcium chloride dihydrate, disodium phosphate dihydrate and Trizma Base were purchased from Sigma Aldrich (Steinheim, Germany).

### 4.2. Methods

#### 4.2.1. Synthesis of Oxidized Pullulan (OP)

OP was obtained following the method proposed by Bruneel and Schacht [30]. Briefly, pullulan in an aqueous solution was oxidized with sodium periodate in a 3:1 molar ratio IO_4_^−^: GU (glucopyranose unit of pullulan). The reaction took place under mild stirring for 6 h in the dark at room temperature followed by the addition of ethylene glycol in a 1:1 molar ratio relative to NaIO_4_ to inactivate the unreacted periodate. The reaction product was dialyzed against water for 48 h. The OP was recovered using freeze-drying, and the yield was 80%. The content of the aldehyde groups of OP was determined using potentiometric titration using the hydroxylamine method [103], and the degree of oxidation was 30% (3.625 meq./g dialdehyde pullulan). The viscosity-average molecular weight (Mv) of OP, determined by the viscometric method [104], was 12,000 g/mol.

#### 4.2.2. Preparation of the Tri-Component Hydrogels

CS was solved in 0.1 M HCl to obtain a 2.5% (*w*/*v*) concentration. The pH of the viscous solution was slowly increased to 6 with 0.1 M NaOH, close to the pKa value of CS to obtain unionized –NH_2_ groups able to react with aldehyde groups of OP. The OP solution (10%, *w*/*v*) was obtained under mild stirring overnight and brought to pH = 6. PVA solution (10%, *w*/*v*) was obtained by dissolving the polymer at 90 °C for six hours. The first step in the preparation of the hydrogels consisted of mixing the CS and PVA solutions; then, water was added so that the final precursor solution contained 1% CS. The concentration of PVA in the precursor solution was 1% or 1.5%. The PVA/CS mixture solution was added into cylindrical molds with a 10 mm diameter. A certain amount of 10% OP solution was slowly added to each mold under mild stirring to ensure a 1:1 or 1:1.5 molar ratio between CS and OP and a final solution volume of 2 mL. Due to the chemical cross-linking between amino groups and aldehyde groups, the formation of a viscous gel was observed after 1–2 min. After three hours at room temperature, the hydrogels were subjected to six F-T cycles (18 h at −20 °C and 6 h at room temperature) to favor the physical cross-linking, and then lyophilized at −0.05 mBar and −50 °C. To remove the unreacted polymers and the NaOH excess, the hydrogels were purified by washing with distilled water until the conductivity of the filtrate was 2 µS/cm, and then dried using lyophilization. Gel fraction (GF) was calculated as a ratio between the weight of the hydrogels after the purification step and lyophilization and the weight of the dried samples before the purification [40].

#### 4.2.3. Mineralization of the Hydrogels Using Alternate Soaking Method

The dried hydrogel discs (around 70 mg) were first swollen in water, then individually immersed in 10 mL of 0.12 M Na_2_HPO_4_ solution (pH = 8.6) for two hours, followed by intensively washing with distilled water to remove the excess of phosphate solution. Then, the hydrogels were immersed for two hours in 10 mL of 0.2 M CaCl_2_/0.5 M Trizma base (pH = 7.4), followed again by intensive washing with distilled water as previously described. The alternative soaking was repeated seven times over five days in fresh solutions, following a pre-established protocol in which, during the night, the hydrogels remained immersed either in calcium or in phosphate solution, in a successive order. After the final washing step, the hydrogels were recovered using freeze-drying for 48 h and the scaffolds were characterized.

#### 4.2.4. CaP Formation in the Absence of Hydrogels

In a glass vial, 10 mL of 0.2 M CaCl_2_/0.5 M Trizma base was added over 10 mL of 0.12 M Na_2_HPO_4_ solution, and a white precipitate was instantaneously formed. The precipitate was kept in mother solutions for maturation for five days at room temperature and then rigorously washed with distilled water. The white precipitate was frozen for 12 h and then freeze-dried for 48 h.

#### 4.2.5. Physico-Chemical Characterization of the Un-Mineralized and Mineralized Hydrogels

The CS content in hydrogels was quantified by two methods of analysis: ninhydrin assay (to determine the free amino group content) and by an analytical method that uses catalytic high-temperature combustion and flow injection (to determine the total nitrogen content). For the ninhydrin assay, 5 mg of hydrogel was swollen in 5 mL 0.5% acetic acid for 12 h in a test tube, and then 5 mL freshly prepared ninhydrin reagent was added [105]. The mixture was heated in a water bath at around 100 °C for 30 min to ensure the interaction between -NH_2_ of CS with ninhydrin reagent until a dark violet condensation product was obtained. The solution was cooled down, and 0.5 mL was taken and mixed with 10 mL ethanol/water 1/1 [41]. The absorbance was read at 570 nm using an Evolution 201 UV–Visible Spectrometer (Thermo Fisher Scientific, Waltham, MA, USA). A calibration curve for glycine was previously obtained for the quantification of the free -NH_2_ groups.

To determine the total nitrogen content, the hydrogels were first subjected to acid hydrolysis following the method described in the literature [106]. The solutions were diluted according to the instructions in the device manual and analyzed using a Multi N/C 3100 pharma—TOC/TN device (Analytik Jena, Germany). The cross-linking degree (*C.D.*) of the CS chains from the hydrogels was estimated from the total nitrogen content and free amino groups using Equation (1):

(1) $$C.D.=\left(\frac{N_{HG}}{N_{CS}}\times E_{CS}-E_{HG}\right)/\left(\frac{N_{HG}}{N_{CS}}\times E_{CS}\right)\times 100$$

where *N_HG_* and *N_CS_* are the nitrogen contents in hydrogel and chitosan (mg/g), *E_CS_* is the cationic charge density of CS (4.735 meq. NH_2_ groups/g CS), and *E_HG_* is the cationic charge density of the hydrogel (meq. NH_2_/g hydrogel) determined from the ninhydrin test.

The PVA content was determined as the remaining residue resulted from the acid hydrolysis previously mentioned.

Fourier-transform infrared spectroscopy (FT-IR) spectra were recorded using an FTIR Bruker Vertex 70 spectrometer (Bruker, Vienna, Austria), in the range of 4000 ÷ 400 cm^−1^, using KBr pellet technique. TOPUS 6.5 software was used for the FTIR data processing. For the deconvolution of the spectra, OriginPro 8.5.0 SR1 (OriginLab Corporation, Northampton, MA, USA) software was used, by applying Gauss nonlinear curve fit for un-mineralized hydrogels and Lorentz nonlinear curve fit for CaP and the mineralized CS_1_PVA_1_OP_1_-CaP hydrogel, respectively.

Thermogravimetric analysis was performed on a Discovery TGA 5500 (TA Instruments, New Castle, DE, USA) at a heating rate of 10 °C/min. Samples of 6.2 mg weight placed in platinum crucibles were heated from room temperature up to 700 °C under a flow of N_2_ (25 mL/min).

The surface morphology of the hydrogels without CaP was investigated using an Environmental Scanning Electron Microscope type Quanta 200 (FEI Company, Hillsboro, OR, USA), operating at 20 kV with secondary electrons. The mineralized hydrogels were coated with 10 nm platinum at 30 mA using a Leica EM ACE200 Sputter coater and the surface morphology was examined using a Verios G4 UC Scanning Electron Microscope (Thermo Scientific, Bruno, Czech Republic). The energy-dispersive X-ray analysis (EDX) was also performed on the same samples using an EDX analyzer Octane Elect Super SDD detector, and the Ca/P ratio was then calculated.

X-ray diffraction (XRD) patterns were obtained using a Rigaku Miniflex 600 diffractometer (Rigaku, Tokyo, Japan) with monochromated Cu-Kα radiation (λ = 1.5406 Å wavelength, 15 mA emission current and a 40 kV voltage). Powder diffraction patterns were captured in the 4–60° range, with a step of 0.01° at 2°/min scan speed. SmartLab II v.4 software was used for background reduction, smoothing, and WPPF (Whole Powder Pattern Fitting) refining, while the powder patterns were determined using the Crystallography Open Database (COD).

The swelling ratios (*SR*) of the hydrogels without and with CaP were calculated to evaluate their capacity to swell in physiological conditions (pH = 7.4 at 37 °C). Discs of dried hydrogels were immersed in phosphate buffer (PB) pH 7.4 (0.05 M NaH_2_PO_4_ + Na_2_HPO_4_) and at different time intervals were withdrawn; the excess of superficial water was removed with blotting paper and they then weighed. *SR* was calculated according to Equation (2):

(2) $$SR=\frac{Ws-Wd}{Wd}$$

where *Ws* and *Wd* are the weight of the swollen and dry hydrogel, respectively.

Degradation of the un-mineralized and mineralized hydrogels was investigated following the method described in the literature [107]. Briefly, the variation of the weight loss of the samples after immersion in phosphate buffer with pH 7.4 at 37 °C for 3, 7, 14, and 21 days was calculated using Equation (3):

(3) $$Weight\;loss\;\left(\%\right)=\left(\frac{W_{1}-W_{2}}{W_{1}}\right)\times 100$$

where *W*_1_ and *W*_2_ are the weight of the dry mineralized hydrogel before and after immersion in PB.

The mechanical properties of the un-mineralized and mineralized hydrogels were investigated using a Texture Analyzer (Brookfield Texture PRO CT3^®^, Brookfield Engineering Laboratories Inc., Middleborough, MA, USA), following the ASTM D882 standard. The cylindrical shape hydrogels were hydrated for two hours in PB pH = 7.4 and then were measured with a digital caliper (~15 mm diameter and 7 mm height). The hydrogels were compressed between two parallel plates with a compression rate of 0.2 mm/s up to 70% deformation. The strain (*ε*) and the compressive nominal stress (*σ*) were calculated using Equations (4) and (5):

(4) $$\varepsilon =∆l/l_{0}$$

(5) σ=F/A

where *l*_0_ is the initial length, Δ*l* is the change in length, and *F* is the force (N) acting perpendicular to the area *A* (m^2^) of the sample. The elastic modulus, E, was calculated from the slope of the strain–stress curve at the initial linear segment (0.1–10% strain). The experiments were performed in three replicates.

#### 4.2.6. Cytocompatibility and Cytotoxicity of the Un-Mineralized and Mineralized Hydrogels

The cytocompatibility of hydrogels without and with CaP was performed using the human osteosarcoma-derived osteoblast cell line MG-63 (American Type Culture Collection, ATCC) (Manassas, VA, USA). Osteoblasts were maintained in Dulbecco’s modified Eagle’s medium—1 g/L glucose (SIGMA-Aldrich, Merck KGaA, Darmstadt, Germany), supplemented with 10% fetal bovine serum and 100 U/mL penicillin/streptomycin at 37 °C and 5% CO_2_ in an incubator.

Hydrogels were cut into 2 mm × 2 mm × 1 mm pieces using a stereomicroscope (Zeiss, Oberkochen, Germany) and UV-sterilized for 6 min in 24-well plates. Hydrogels were seeded with 10 µL MG-63 cell suspensions with different densities depending on the retention time of the cells in 3D culture: 120,000 cells/hydrogel for 24 h, 70,000 cells/hydrogel for 7 days, 40,000 cells/hydrogel for 14 days, and 20,000 cells/hydrogel for 21 days. The experiment was conducted in duplicate for each sample. Ten minutes after adding the cell suspension (to ensure cell penetration and attachment to the hydrogel fibers), 0.5 mL complete culture medium was added to each well. In parallel, osteoblasts were seeded in a two-dimensional (2D, monolayer) culture model at the above-mentioned densities for each experimental time. The medium was retrieved and replaced every 2–3 days and kept at −20 °C for further determinations.

To assess the cellular morphology of osteoblasts, cells cultured in 2D and those cultured in hydrogels were fluorescently labeled with phalloidin and 4′,6-diamidino-2-phenylindole (DAPI) to highlight F-actin filaments and nuclei, respectively. At each experimental time, the samples were washed with warm 1× PBS three times, fixed with 4% paraformaldehyde (PFA) in 0.1 M phosphate buffer (pH 7.4) for 20 min at room temperature and permeabilized with 0.2% TritonX-100 for 5 min at room temperature. Phalloidin labeling was performed at room temperature for 40 min by incubating the samples with 200 ng/mL phalloidin-TRITC (cat. no. RD-5783, R&D Systems, Minneapolis, MN, USA). After removal of unbound dye by 3 successive washes with PBS, nuclei were labeled with 1 µg/mL DAPI for 10 min at room temperature. Fluorescence image acquisition (3 fields/sample) was performed using the 20× objective of an Olympus IX81 inverted microscope (Olympus Corporation, Shinjuku, Tokyo, Japan) and the Z-stacking function of CellSens Dimension 1.5 software was used for the 3D culture model. Consecutive images of Z-stacks were obtained at a 10 µm interval using detection filters for TRITC (red, phalloidin-TRITC) and DAPI (blue) and reconstructed in ImageJ software version 1.8.0 developed at the National Institutes of Health (NIH), USA.

The cytotoxicity of the hydrogels was assessed by labeling MG-63 osteoblasts with calcein/propidium iodide using the Live/Dead kit (SIGMA-Aldrich, Merck KGaA, Darmstadt, Germany) according to the manufacturer’s instructions. At different time intervals (24 h, 7 days, 14 days and 21 days), 2D and 3D cultured cells were incubated with the kit reagents for 30 min in the dark. Fluorescence images (3 fields/probe) were acquired using the 10× objective and using FITC (green, calcein) and TRITC (red, propidium iodide) detection filters to highlight viable and dead cells, respectively. In the case of hydrogels, Z-stacks were made at an interval of 10 µm. Images were processed in ImageJ 1.42q software (U. S. National Institutes of Health, Bethesda, MD, USA), and the average fluorescence of the two channels was quantified using ImageJ and expressed as a percentage ratio.

The toxicity of the hydrogels was also assessed by quantifying the release of adenylate kinase (AK) into the culture medium after cell death using the ToxiLight™ BioAssay Kit (Lonza Bioscience, Basel, Switzerland). Luminescence measurements were made at one second using a Mithras LB 940 Multimode Microplate Reader luminometer (Berthold Technologies GmbH & Co. KG, Oak Ridge, TN, USA). Data were normalized to the value obtained on the 2D culture model at 24 h, considered 1, and expressed as the mean ± S.D. (standard deviation) of an experiment performed in duplicate.

### 4.3. Statistics

Data are expressed as mean ± standard deviation (S.D.) of at least two independent experiments performed in duplicates. Comparisons between groups were performed using a two-tailed Student *t*-test using GraphPadTM Prism software version 9.2.0 (GraphPad Software, La Jolla, CA, USA).

## Figures and Tables

**Table 1:** Composition of polymers (CS/PVA/OP) in the initial mixture and in hydrogels.

	Initial Mixture			Final Composition of Hydrogels				
Sample Code	CS:PVA Mass Ratio	NH_2_:CHO Molar Ratio	CS/PVA/OP (wt. %)	GF (wt. %)	N (mg/g) ^a^	NH_2_ (meq./g) ^b^	C.D. ^c^ (%)	CS/PVA/OP (wt. %) ^d^
CS_1_/PVA_1_/OP_1_	1:1	1:1	29.4/29.4/41.2	61 ± 4.8	29.95 ± 0.22	0.68 ± 0.01	67 ± 1.7	43.7/32.7/23.6
CS_1_/PVA_1_/OP_1.5_	1:1	1:1.5	24.4/24.4/51.2	64 ± 1.4	30.88 ± 0.26	0.59 ± 0.02	73 ± 0.8	45.0/19.6/35.4
CS_1_/PVA_1.5_/OP_1_	1:1.5	1:1	25.6/38.5/35.9	67 ± 3.9	24.75 ± 0.1	0.49 ± 0.01	72 ± 1.1	36.1/42.6/21.3

^a^ calculated from total nitrogen content; ^b^ calculated using the ninhydrin test; ^c^ cross-linking degree calculated from the content of free amine groups determined by the ninhydrin test and from total CS content determined from nitrogen content in hydrogels using Equation (1); ^d^ CS was calculated from nitrogen content and PVA was determined as a solid residue after acidic hydrolysis.

**Table 2:** Composition of mineralized hydrogels and their mechanical properties in comparison with the pristine hydrogels.

Sample Code	Ca/P Mass Ratio from EDX	CaP Determined from TGA (wt. %)	HAp:DCPD (wt. %) from XRD	Young′s Modulus (kPa)	Compressive Nominal Stress at 70% Deformation (kPa)
CS_1_/PVA_1_/OP_1_	-	-	-	6.5 ± 0.7	10.7 ± 1.5
CS_1_/PVA_1_/OP_1.5_	-	-	-	6.6 ± 0.5	7.3 ± 0.1
CS_1_/PVA_1.5_/OP_1_	-	-	-	9.3 ± 0.4	13.4 ± 0.2
CS_1_/PVA_1_/OP_1_-CaP	1.84 ± 0.04	63.9	79:21	70.4 ± 11.6	50.6 ± 4.9
CS_1_/PVA_1_/OP_1.5_-CaP	1.77 ± 0.09	62.6	89:11	101.8 ± 4.2	64.3 ± 2.5
CS_1_/PVA_1.5_/OP_1_-CaP	1.62 ± 0.15	62.8	91.5:8.5	117.3 ± 5.2	81.4 ± 5.1

DCPD—non-apatitic phase.

**Table 3:** XRD powder quantitative analysis of CaP and mineralized hydrogels using WPPF.

Hydrogel	CaP		CS_1_/PVA_1_/OP_1_-CaP		CS_1_/PVA_1_/OP_1.5_-CaP		CS_1_/PVA_1.5_/OP_1_-CaP	
Phase Name	HAp	DCPD	HAp	DCPD	HAp	DCPD	HAp	DCPD
Weight Fraction, wt. %	71.7	28.3	79	21	89	11	91.5	8.5
a (Å)	9.55	6.51	9.44	6.90	9.30	6.51	9.44	6.39
b (Å)	9.55	14.96	9.44	20.15	9.30	15.77	9.44	15.14
c (Å)	6.86	5.27	6.87	5.59	6.70	6.21	6.87	5.81
α (º)	90	90	90	90	90	90	90	90
β (º)	90	118.19	90	118.10	90	121.60	90	119.08
γ (º)	120	90	120	90	120	90	120	90
V, (Å3)	541.11	452.33	529.67	686.23	502.16	542.66	529.41	490.83
Cs, (Å)	134	64	131	50	260.9	49	167	52
C, (%)	99.31	59.4	100	12	34.50	34.40	48.40	40.80
S (GOF)	1.1003		1.0868		1.0585		1.0252	
Rwp, (%)	3.17		3.89		3.91		3.83	
χ2	1.0519		1.1811		1.1204		1.0511	
COD—Crystallography Open Database	Hydroxylapatite (HAp)-Ca_10_(PO_4_)_6_(OH)_2_ *9002214*				Brushite (DCPD)-Ca(HPO_4_)(H_2_O)_2_ *1533075*			

S—“goodness-of-fit”, which should be close to 1.0 at the end of refinement; Cs—crystallite size; C—crystallinity; V—Lattice Volume.

## Data Availability

Data are openly available in the article.
